# A community based, bottom-up, multi-pronged, technology integrated approach to enhance tuberculosis related awareness and treatment adherence in Uganda: The ACTS model

**DOI:** 10.1371/journal.pone.0318174

**Published:** 2025-02-18

**Authors:** Stavia Turyahabwe, Srikrishna Sulgodu Ramachandra, Subhi Quraishi, Ilmana Fasih, Hilmi Quraishi, Palavardhan Peddapalegani, Akram Ahmad

**Affiliations:** 1 Uganda National Tuberculosis and Leprosy Program, Kampala, Uganda; 2 Uganda Tuberculosis Implementation Research Consortium, Kampala, Uganda; 3 Qaff Africa Foundation, Kampala, Uganda; 4 Department of Community Medicine, KMC Medical College and Hospital, Maharajganj, Uttar Pradesh, India; 5 S. V. University, Tirupati, India; Makerere University College of Natural Sciences, UGANDA

## Abstract

**Background:**

Tuberculosis (TB) is still a major public health challenge globally and Uganda is one among the top 30 high TB burden countries. One of the key factors determining TB treatment success rates and thereby Cure Rates is the adherence to TB treatment, which is still a major challenge globally. WHO DOTS (Directly Observed Treatment Short course) strategy has several limitations and WHO End TB Strategy 2017 suggests a suite of new interventions to improve adherence.

**Objectives of the study:**

a. To present the development and design of ZMQ’s Active Care and Treatment Strategy (ACTS) Model.
b. To present the results of a pilot study done using the ACTS Model.
c. To compare Treatment Adherence Rates between DOTS and Video Observed Treatment (VOT) in the four districts of Uganda.

**Materials and methods:**

Includes presenting the ACTS Model, a pilot study to assess the AGB and ACF by way of a pre-post (Quasi-experimental) study (n = 1000) to assess the impact of AGB exercises, Focus Group Discussions (FGD) to get insights into factors contributing to treatment non-adherence and a comparison between VOT (n = 800) vs. DOTS for treatment adherence.

**Results:**

There was a significant improvement (*p*<0.01) in knowledge and awareness levels of community members post the AGB exercises which included creating awareness using digital storytelling, house visits and more. TB treatment adherence rates among TB patients using VOT was significantly better (*p*<0.01) than those using DOTS.

**Conclusion:**

AGB and ACF play a significant role in creating more awareness amongst the community members and identifying more number of cases. It helps in better treatment seeking behaviour, improved treatment rates and treatment adherence rates and in turn better cure rates. VOT is far more superior to DOTS, as a strategy for TB treatment adherence and VOT decreases the resources required in terms of human resource, time and money and is also a more sustainable mode of treatment adherence.

## Background & review of literature

Tuberculosis (TB) is undoubtedly a major global public health challenge, for which, a permanent and all-faceted solution is yet to come [1]. Globally, TB is one of the top 10 causes of death & the leading cause of death due to infections [2,3]. In 2017, an estimated 10 million people developed TB & 1.6 million died due to the disease [4]. The global number of deaths officially classified as caused by TB in 2023 was 1.25 million and it was almost double the number caused by HIV/AIDS (0.63 million). TB mortality was much more severely affected by the Covid-19 pandemic than HIV/AIDS [3].

A 2017 World Health Organisation (WHO) Fact Sheet highlights that, out of an estimated 9.6 million TB patients living worldwide, 2.7 million live in the African region, making them one of the highest TB burden areas of the world [5]. Three out of four people infected with HIV & TB, live in Africa [3]. The burden of HIV associated with TB is highest in African region and Uganda is one among the 30 high TB burden countries globally [3].

TB incidence rate in Uganda stands at around 200 cases per 100,000 population [6]. Uganda has around 96,000 new cases of tuberculosis (TB) a year and 32% of them being HIV infected, making it one among the 30 high burden countries for Tuberculosis and TB/HIV co-infection globally [7,8]. Only about half of these cases (i.e. around 45,000 TB patients), receive treatment and it is estimated that, one in four TB patients in Uganda are not adherent to their TB treatment [9]. Around 25,000 Ugandans die of TB or TB + HIV each year [3].

There have been several milestone achievements in tackling Tuberculosis, one of which being the Directly Observed Treatment Short Course or popularly known as the DOTS strategy. The DOTS Strategy improved treatment adherence and thereby treatment completion rates and treatment success rates to a great extent. But it still has several challenges, which include, being human resource intensive, dependency on an external individual and being cost and time intensive [10]. Multiple trials on DOTS have failed to demonstrate improvement in treatment outcomes using DOTS [10]. Some of the major hurdles and challenges faced by TB patients using DOTS include, loss of job and livelihood as a result of daily reporting to the health observer/DOT provider where DOTS is being implemented, lack of adequate information on medication adherence, lack of social support and lack of focused attention by Health Care Providers (HCPs) [11–13]. Stigma and discrimination and inhibiting patient autonomy are other important drawbacks [11–13]. There is evidence that DOTS does not perform better than self-administered therapy in cases of disease relapse and acquired drug resistance [14]. Also, Missing Cases and Cases lost to Follow-up is another major problem in controlling / eliminating / eradicating TB. It is partly due to the top-down design of the existing TB case finding strategy and also the passive case finding modes, that case detections happen only when patients develop severe condition of TB and hence the importance of Active Case Finding (ACF) [15].

In Uganda, treatment success rates were reported to be 70–75% [10]. Treatment units use a mix of facility- and community-based approaches to DOT with no clear evidence on adherence levels and DOT implementation other than documentation in the registers that patients are on DOTS. This state of affairs had resulted in the 70% treatment success with over 10% of the patients being lost to follow-up, a performance which is far below the WHO defined treatment success rates of 90% [11]. There are several factors associated with adherence / non-adherence to TB treatment, including, demographics, geography, lack of adequate knowledge and economic related, to just name a few [16–18]. TB usually affects people who are hard to reach such as poor, unemployed and homeless, those lacking social support networks and unstable living circumstances. Factors like overcrowding, lack of proper ventilation in living spaces, lack of healthy nutrition, etc. also play a major role in determining one’s vulnerability to TB [12–14,19]. However, in the recent years, the treatment success rates have been improving in Uganda, with a reported treatment success rate of above 90% for the first time in 2023–2024 [20].

Although TB is successfully treatable if the treatment begins promptly, follows the correct regimen, and remains uninterrupted for the full 6 to 9 months’ course, there are notable challenges in achieving this outcome. Factors such as a high default rate, treatment interruptions, non-adherence to therapy, and insufficient understanding of the disease, significantly contribute to suboptimal TB treatment outcomes. Consequently, ensuring accurate diagnosis, utilization of effective anti-TB medications, and promoting optimal treatment adherence, become critical strategies for reducing morbidity and mortality associated with TB and curbing its spread within the population. In the recent years, based on advancements in technology and also mobile penetration into all strata of people and in tribal, rural and urban areas, several variants and improvements in the DOTS model of treatment adherence have been tried. Evidence suggests that there is widespread and ever-growing telephone ownership and mobile network coverage in Sub-Saharan Africa [21]. The WHO End TB Strategy 2017 suggests a suite of new interventions to improve adherence, including mobile-phone based short message service (SMS) text messages and digital adherence monitoring mechanisms [22]. A 2018 published study from Uganda indicates that around 75% of TB patients own mobile phones and are willing to receive TB-related communication on their phones [23]. The study also revealed that even patients without mobile phones were willing to receive telephonic communication on a family mobile phone [23].

Qaff Africa Foundation (QAF) is an indigenous Technology for Development, Not-for profit Organization based out of Kampala, Uganda. QAF has designed and implemented its unique Active Care and Treatment Strategy (ACTS) which includes Active Ground Building (AGB), Active Case Finding (ACF) and also Active Patient Compliance using Video Observed Treatment (VOT) amongst other strategies. As part of the South–South Collaboration, QAF has been technically supported by ZMQ Development, an NGO based out of India.

## Objectives of this study

a. To present the development and design of ZMQ’s Active Care and Treatment Strategy (ACTS) Model.
b. To present the results of a pilot study done using the ACTS Model.
c. To compare Treatment Adherence Rates between DOTS and Video Observed Treatment (VOT) in the four districts of Uganda during the time period of May 2021 to November 2022.

## Materials & methods

The Materials and Methods section includes 2 key sub-sections and a brief description of the same:

A. ZMQ ACTS Model and its six key Components and
B. The Pilot study conducted in implementing the ACTS Model.

### Study setting

The pilot study was conducted in the four districts of Kabarole, Mbarara, Kampala and Jinja. In Kampala, the ACTS approach was tested with the vulnerable communities in the urban slums and settlement areas. In Jinja, Mbarara and Kabarole, the study was conducted amongst the vulnerable and low-resource rural communities of the district. All the identified TB suspected cases were referred to the Regional Referral Hospitals (RRH) in case of Jinja, Mbarara and Kabarole and to Mengo and Mulago Hospitals at Kampala. Majority of the subjects of the study were semi-literate and were from a lower socio-economic background. The scope of the study was to test uptake of the ACTS approach amongst these vulnerable communities.

### Ethics and research approval

Ethics and Research approval for the conduct of this study was obtained from the Research Ethics Committee (REC) of Mengo Hospital, which was finally endorsed by the Uganda National Council for Science and Technology (UNCST) with the UNCST research registration approval number: **HS1183ES**.

### Recruitment period for this study and participant consent

Start Date of recruitment period: 2^nd^ May 2021.

End Date of recruitment period: 30^th^ November 2022.

Structured Questionnaires (wherever applicable) were administered by the Health Care Worker (Village Health Teams) after explaining the study to the participant in their own language and after obtaining a Written Informed Consent.

An Informed Written Consent was obtained by each participant that was involved in the Study. The participation in the Study was voluntary. No Minors were involved in the Study.

The Study that we conducted was a Prospective Study and for comparing our Study data to the existing DOTS data from the Government Programme, anonymized patient data was obtained from the National Tuberculosis and Leprosy Programme (NTLP). The anonymized NTLP data was accessed in December 2022.

The project team and the authors had access to information that could identify individual participants during data collection–as this was an Implementation Research project and the TB patients / participants had to be followed up for a period of 6–9 months. But, while doing the analysis of the project data, all data were anonymized and used for analysis purposes.

#### A. ZMQ ACTS model

Qaff Africa Foundation proposes and has implemented a holistic TB awareness building, identification and treatment model called the ACTS Model and has validated this model through a pilot study in 4 districts of Uganda:

In order to combat TB, the Qaff Africa Foundation has come up with the ACTS (Active Care and Treatment Strategy) model. It is a multi-tier system change strategy which uses the innovations in technology as one of the ways to tackle TB. With ubiquity of mobile networks and increasing number of cell-phone users, the technology embedded design offers a system changing approach in creating awareness, case identification, investigations, treatment and overall management of TB. The model includes patients as part of the solution design, which is a Bottom-Up Technology model. It empowers patients to take control of their treatment with limited supervision. It reduces the burden on DOTS providers which is human resource intensive.

The ACTS model has technologies embedded & integrated at different levels & all these technologies comprise the Active Care and Treatment Strategy or the ACTS strategy. Some of the salient features of ACTS are:

- Fully Technology Linked Health and Adherence Management System for TB;
- Creating an Environment for Active Adherence;
- Reducing the burden of existing infrastructure;
- Having an increased Community involvement in adherence.

**Fig 1:**
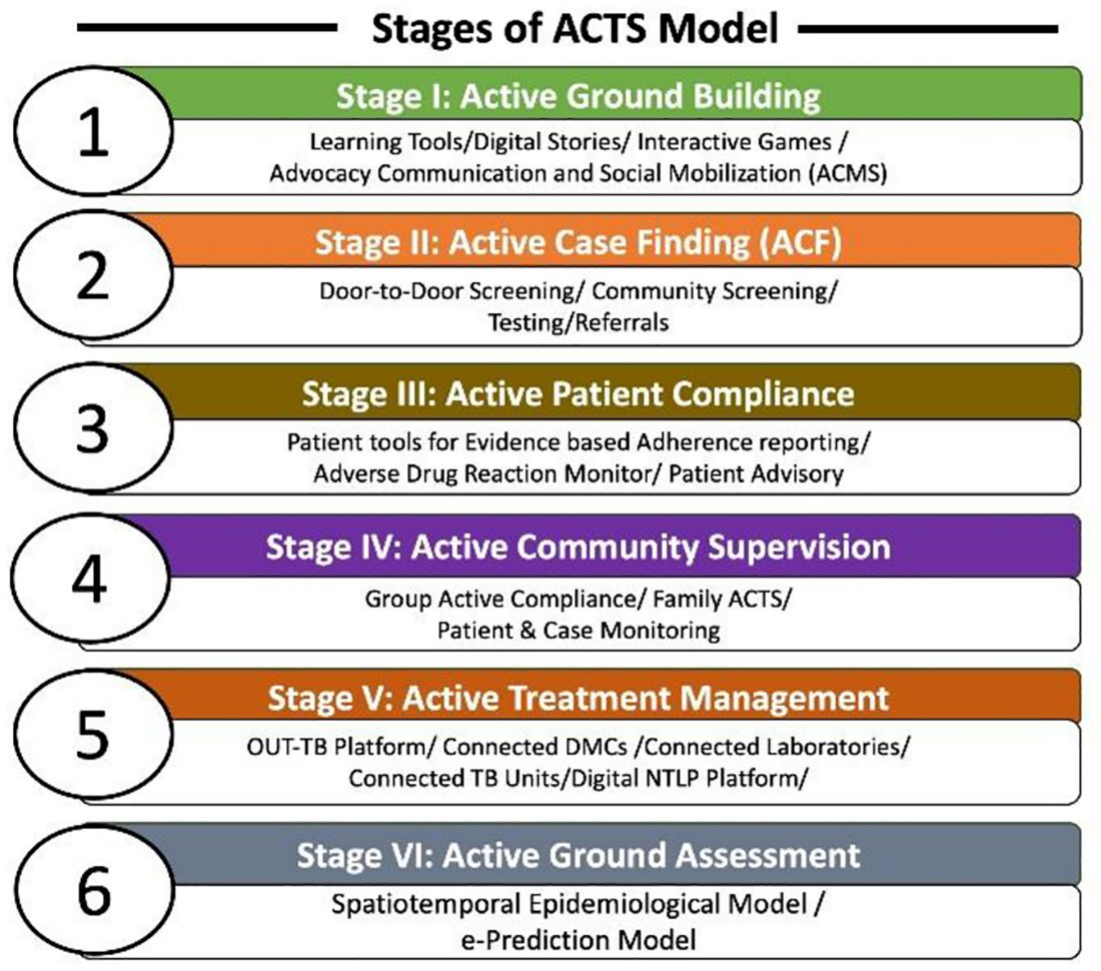
Six components of ACTS—Universal Technology Based TB treatment management solutions. A Community Based, Bottom-Up, Multi-pronged, Technology Integrated Approach to enhance Tuberculosis related Awareness and Treatment Adherence in Uganda: The ACTS Model.

The ACTS model consists of six Components that aim to enhance tuberculosis (TB) treatment and management (Fig 1).

The first four Components focus on the patient and community, aiming to empower them and improve treatment outcomes. The remaining two Components shift the focus to the health system, providing tools and support for efficient TB management. The following are the Components of ACTS:

1. **Active Ground Building**: Social and Behavioural Change Communication (SBCC) approach is used through which all the community members are engaged using interactive games, workshops, mobilization meeting with families & patients using digital-based IEC (Information, Education and Communication) & ICT (Information and Communication Technology) material for creating a basic essential awareness on Tuberculosis. This Component was deployed in the districts of Kabarole and Mbarara in Uganda. The project team visited the sites, coordinating with village heads to gather participants for sessions at specific times. During these sessions, the team used both digital and non-digital tools to teach and engage the participants. Around 1,560 people were reached at each of the two sites. To evaluate the effectiveness of this intervention, a pre–post questionnaire-based survey was conducted with a simple random sampling.
2. **Active Case Finding** uses systematic screening of suspect populations with active TB in the high-risk population. Door-to-door sensitisation and screening using mobile screening and referral tools to all the missed cases using technology-based approaches. The approach provides links with facilities to follow up and collect sputum from presumed patients. This Component was deployed in the districts of Kabarole and Mbarara in Uganda.
3. Mobile phone based **Active Patient Compliance toolkit** is a solution designed for TB patients to report their compliance to treatment by sending real time evidence-based video of swallowing TB medicines (VOT–Video Observed Treatment) from their homes, at workplaces & in communities. The toolkit has other components like patient information, scheduler, test reports and learning zones & is connected with a Real-time dashboard for DOT Providers to monitor each and every patient daily. Our Video Observed Treatment (VOT) stands out from other VOT methods due to its integration with a range of Behaviour Change Communication (BCC) tools. These tools include digital stories on TB, educational modules about TB, and alert messages or reminders designed to assist patients in remembering to take their medication. These additional BCC tools play a pivotal role in enhancing patient knowledge about TB, promoting timely medication adherence, and encouraging the adoption of correct health-seeking behaviours. The Component was implemented in the districts of Kampala, Kabarole, Mbarara and Jinja in Uganda.
4. **Active Community-led Supervision** is the process of actively engaging community members of the TB patients for observing the progress and compliance of treatment of patients. The Community led supervision model is established with patients, who are incapable of self-managing treatment using digital adherence technologies like smart-phones. Essentially such patients are generally young children, elderly, people with no digital literacy and also family members, and members in close community groups. The community supervisors are from the community itself and are mainly TB patients or successfully treated community members. Involving TB patients and former patients makes the patients be at the centre of TB management and programming. This component was implemented in the districts of Kampala, Kabarole, Mbarara and Jinja in Uganda.
5. **Active Treatment Management**: Active Treatment Management is a **Fully-Technology linked Model (F-TLM)** for TB treatment and management using **Open and Universal Technology based TB (OUT-TB)**. OUT-TB framework empowers the community health workers and DOTS providers to manage treatment of patients using technology. Although this framework is lot more dependent on specific technologies used by different community health workers in the area of TB treatment but the larger objective of this framework is to focus on introducing technology at different CHW layers in the TB system such as Volunteers engaged in Active Case Finding, Field Coordinators, Lab Technicians, DOTS Providers and Treatment Supervisors etc.
6. **Active Ground Assessment**: is a model for persistent simulation (continuous data ingestion) and endemic prediction of TB, wherein TB related data is continuously gathered, assimilated and data scrutinized for predicting outbreaks. It is derived based on multiple factors of TB epidemiology and community based social networking parameters. ZMQ’s Active Compliance Treatment System will help generate these predictive models to the level of villages and community mohallas. With the help of such tools, ZMQ aims to design customized interventions to combat Tuberculosis.

#### B. The pilot study to validate the ZMQ ACTS model

The Pilot study adopted a **Mixed Methods Approach**, wherein the **Quantitative data** on awareness created by the ACTS Model was assessed using a **Quasi-Experimental Study Design** by using a structured pre-validated questionnaire (Pre-& Post-Awareness, knowledge testing) (Annexure 1 in S1 File), and the adherence to TB treatment using VOT was compared with the DOTS Adherence data; the **Qualitative data** on the reasons related to low initial levels of awareness, preference of TB patients for VOT in relation to adherence to treatment, challenges due to the Covid-19 pandemic and how they were able to overcome them, were assessed using **Focus Group Discussions** (Annexure 2 in S2 File).

Out of the six components of the ACTS model, this paper discusses the 4 components that are relevant to the community based activities. These components were implemented in a timely, phase wise manner in the following 4 districts in Uganda:

- Kabarole
- Mbarara
- Kampala
- Jinja

The pilot study: Under the ACTS Model, AGB and ACF was conducted among the vulnerable general population while, the components of Active Patient Compliance Tool Kit, Active Community-led Supervision and Active Treatment Management were done amongst a representative sample of the vulnerable patient population. Active Ground Assessment was conducted in the final stage of the pilot study, but as part of the programme, this would be a dynamic ongoing process.

The ACF and AGB interventions were undertaken between January 2022 and June 2022 (6 months) and Active Patient Compliance intervention from May 2021 to November 2022 (18 months) in Uganda.

### Pilot study: Sample size and duration

Using the formula: n = Z^2^(P)(1-P)/e^2^ for sample size calculation, where P is the prevalence of TB (we have taken it at a higher side of 50 percent) = (1.96)^2^(0.5)(1–0.5)/(0.05)^2^, a sample size of 385 per site was obtained. However, a larger sample size of 500 per site, was considered for the Study. This was to account for potential issues of non-response, missing data, outliers, ensuring sufficient power and representativeness. This adjustment provided greater precision in estimates and robustness of the analysis, enhancing the validity of our study findings. The details of the various sites, sample size and duration of the study is given below (Table 1).

**Table 1:** Sample size and duration of the pilot study.

Stages	Site	Sample Size	Time period
Active Ground Building	Kabarole Mbarara	500 from each site	January 2022 to June 2022
Active Case Finding	Kabarole Mbarara	500 referral cases from each sites	January 2022 to June 2022
Active Patient Compliance Also known as SAC (Self Active Compliance)	Kampala Kabarole Mbarara Jinja	200 VOT patients from each of the 4 sites = total 800 patients	January 2021 to June 2022
Active Community-Led Supervision Also known as GAC (Group Active Compliance)	Kampala Kabarole Mbarara Jinja	50 VOT patients from each of the 4 sites = total 200 patients	January 2021 to June 2022

#### Exclusion and inclusion criteria

There were no specific inclusion or exclusion criteria for AGB and ACF–as awareness activities and case finding was done amongst the adult general population.

The inclusion criteria for Active Patient Compliance and Active Community Led Supervision:

a. Patients aged 18 years and above were considered;
b. Both Pulmonary and extra-pulmonary Drug Sensitive TB patients;
c. Patients enrolled during the period of May 2021 till November 2022 within 1 month of treatment initiation, and in the intensive phase.

Active Ground Building and Active Case Finding interventions under the ACTS Model were piloted for a period of 6 months (January—June 2022) at Kabarole & Mbarara districts of Uganda and simultaneously data were collected at 2 different times: a. As a baseline, before implementing the different interventions of the ACTS Model & b. As a part of post-implementation. (Endline survey).

The data collection included both quantitative and qualitative data. The data on VOT was collected for a period of 18 months (May 2021—November 2022).

### Qualitative data collection

Focus group discussions (FGDs) were conducted to gain insights into the level of knowledge, attitudes, and perceptions of the local community regarding TB and its treatment adherence, impact of Covid-19 pandemic on their livelihood in general and on TB treatment seeking and challenges in relation to the same. The information gathered from the FGDs were analysed using Content Analysis to identify common themes and sub-themes related to TB in the local community.

## Analysis of data

### A. Quantitative data

The data was analysed using statistical software SPSS version 23.

Active Ground Building activities were conducted from January 2022 to June 2022 in two districts namely Kabarole and Mbarara. Between this time period, 1560 community people were reached in each district, through community awareness sessions. Out of the 1560, 500 participants were randomly selected for pre and post assessment at each of the sites.

A Paired t-test was used to assess the knowledge levels of participants, before and after conducting the AGB Activities.

### B. Qualitative data: Insights from the focus group discussions

The ground team conducted FGDs in two districts–Kabarole and Mbarara. A total of 8 FGDs were organized in each district (i.e. a total of 16 FGDs and each FGD had 7–8 participants). In each district, 2 FGDs were conducted with exclusive male participants and 2 FGDs with exclusive female participants. This was done to ensure full and stress-free participation of the participants, considering the fact that Tuberculosis is still being associated with stigma and discrimination. The total number of participants in Kabarole were 60 & in Mbarara it was 64. The discussions were conducted in the local language, and were then translated verbatim into English for analysis. So, a total of 124 participants were included in the FGDs and valuable insights were obtained as part of these focus group discussions.

Content Analysis of the qualitative data obtained from the FGDs was done. The transcripts were reviewed and coded, with themes emerging from the data. The data analysis helped identify key themes related to TB management, including knowledge and awareness about TB, symptoms, transmission, treatment, myths & misconceptions, and challenges faced by the community in accessing and completing TB treatment.

## Results

### A. From the quantitative data analysis (Table 2)

**Fig 2:**
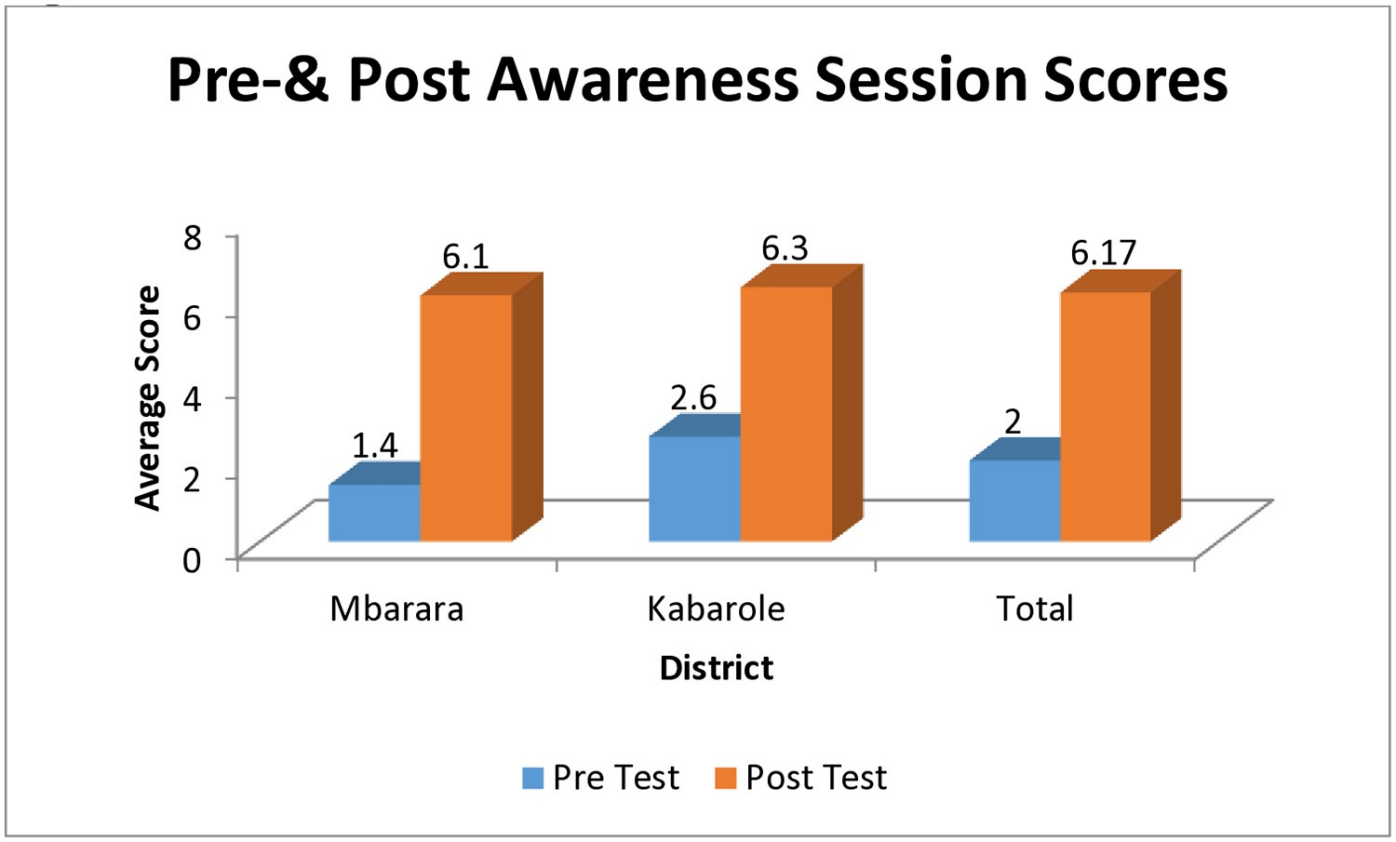
Pre- & post awareness session scores.

The awareness about Tuberculosis and knowledge levels regarding prevention and treatment of Tuberculosis (Table 2) was significantly better during post-test as compared to pre-test and this is attributable to the AGB exercises done by the ground team (awareness sessions, digital story telling tools etc.) **(*p* value ≤ 0.01)** (Fig 2).

**Table 2:** Active ground building: Awareness created about tuberculosis—Pre & post awareness session results (N = 500).

District	Average Score	Mean ± S.D.	t-value	*p*-value
Mbarara	Pre-Test	1.4 ± 1.1	60.420	.001
	Post-Test	6.1 ±1.5		
Kabarole	Pre-Test	2.6 ± 1.1	50.966	.006
	Post-Test	6.3 ± 1.2		
Combined Average	Pre-Test	2.0 ± 0.8	75.475	.0001

#### Active case finding

A house-to-house and community based active TB screening was conducted in the Kabarole and Mbarara regions from January 2022 to June 2022 (Table 3). In Mbarara, a total of 7034 individuals underwent the screening through house-house and community-based sessions. And out of that 9% of screened population (644 individuals) were found as presumptive TB cases and referred to NTLP for confirmatory testing. Out of the 644 referred individuals, 6.99% (45) were confirmed to be positive cases for tuberculosis following further diagnostic tests. Similarly, in Kabarole, a total of 9876 individuals participated in the screening exercise through house-house and community-based sessions. 884 (8.95%) individuals were identified as presumptive TB cases out of 9876 during the screening process. Out of the 884 referred individuals, 8.2% (72) were confirmed to have active TB.

**Table 3:** Details of active case finding by the ground team at Kabarole & Mbarara regions of Uganda.

Parameters		Mbarara	%	Kabarole	%
Screened population	Total	7034		9876	
	Female	3435	49%	4678	47%
	Male	3599	51%	5198	53%
Suspected cases	Total	643	9%	884	9%
	Female	344	53%	399	45%
	Male	309	48%	486	55%
Positive cases	Total	45	6.99%	72	8.2%
	Female	18	40%	26	36%
	Male	27	60%	46	64%

#### Active patient compliance

Two treatment adherence modes were provided to TB patients in the four districts of Uganda namely Kampala, Kabarole, Mbarara and Jinja. A fixed set of patients were randomly selected for this study and their treatments monitored throughout the Intensive phase and then switched them into DOTS mode.

**Table:** 

Total cases in Mbarara for period of January 2022 to December 2022 (t) is 400 (Full coverage of Mbarara North Division)	Total cases in Kabarole for period of January 2022 to December 2022 (t) is 336 (Full coverage of Fort Portal Central Division)
QAFF’s ACF from period of January 2022 to June 2022 (t/2) is 45 (65% coverage of Mbarara North Division)	QAFF’s ACF from period of January 2022 to June 2022 (t/2) is 72 (Full coverage of Fort Portal Central Division)
This implies that QAFF’s ACF contribution is 38.89%	This implies that QAFF’s ACF contribution is 42.85%

The teams conducted ACF activities in the communities of Mbarara and Kabarole Districts for a period of 6 months: Jan to June 2022. ACF activities in Mbarara covered 65% of Mbarara Northern Division contributing to additionality of 45 new cases in a total of 200 estimated patients (based on total patient load of 400 cases in Mbarara for period (t) from Jan 2022 to Dec 2022). In Kabarole District, ACF activities covered almost 100% Fort Portal Central Division in finding 72 new missing cases in a total estimated 168 for the period (based on total patient load of 336 cases in Kabarole for period (t) from Jan 2022 to Dec 2022). In both the districts, QAFF’s ACF contributed to almost 40% of the total patient burden. As a rough estimate, if through the QAFF’s ACTS model approach, tools such as TB related learning toolkit, TB digital stories, TB self-screening tools etc., are made available to all mobile users, it will very likely lead to identification of more cases that were previously overlooked and it could go a long way in identifying over 40% of the missing cases out there in the community.

#### Direct Observed Treatment Short Course (DOTS) Vs Video Observed Treatment (VOT)

Active Patient Compliance & Active Community-led Supervision were piloted among TB patients that were on treatment.

**Fig 3:**
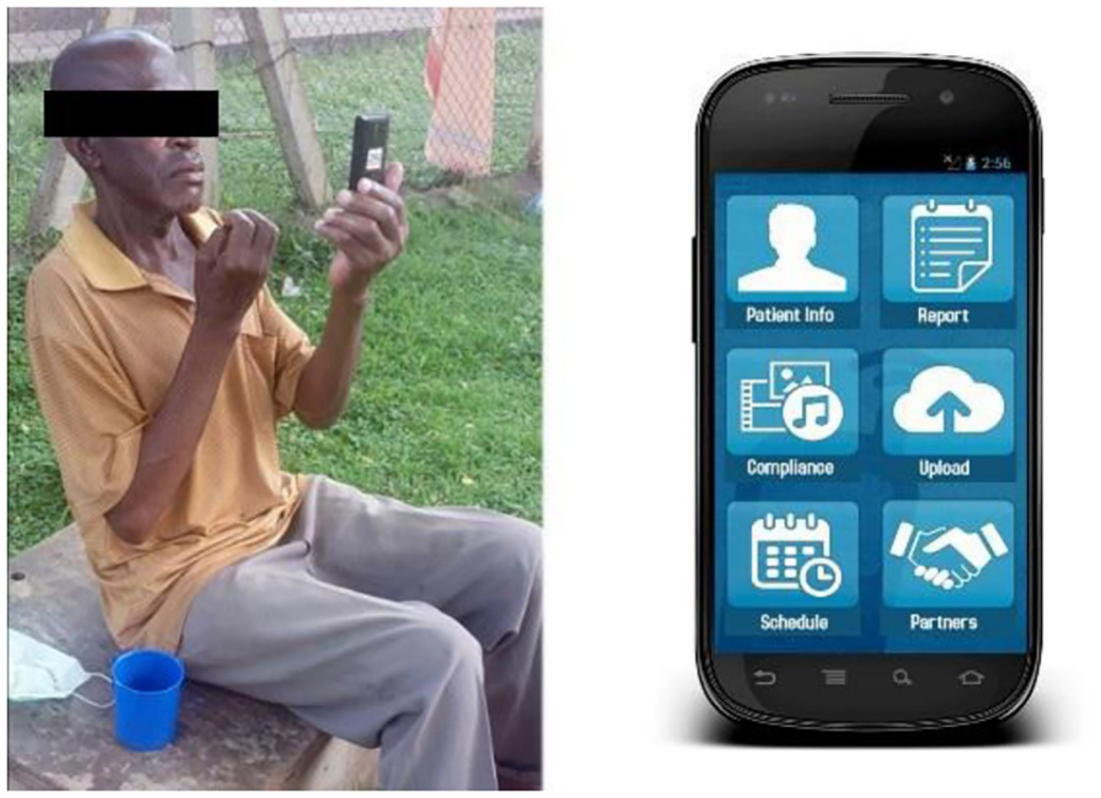
A patient swallowing a tablet using VOT adherence.

QAFF had introduced VOT adherence technology across various districts in Uganda (Fig 3). A random selection of four districts was done, considering diverse geographical locations. Within each selected district, 200 patients of self-active compliance and 50 patients of group active compliance were randomly selected from the existing VOT patient database.

#### Overall TB treatment adherence: DOTS vs VOT (Fig 4)

The treatment adherence rates among Tuberculosis patients were compared between the Direct Observed Treatment Short Course (DOTS) and Video Observed Treatment (VOT) adherence modalities. Using a Chi-Square test, a *p*-value ≤ 0.05 was obtained signifying the fact that, VOT was a significantly better adherence mechanism among TB patients as compared to DOTS in relation to number of TB patients completing the treatment and also getting cured. Similarly the number of TB patients that died due to TB was lesser among those that followed VOT adherence mechanism as compared to those that followed DOTS (Tables 4 & 5).

**Fig 4:**
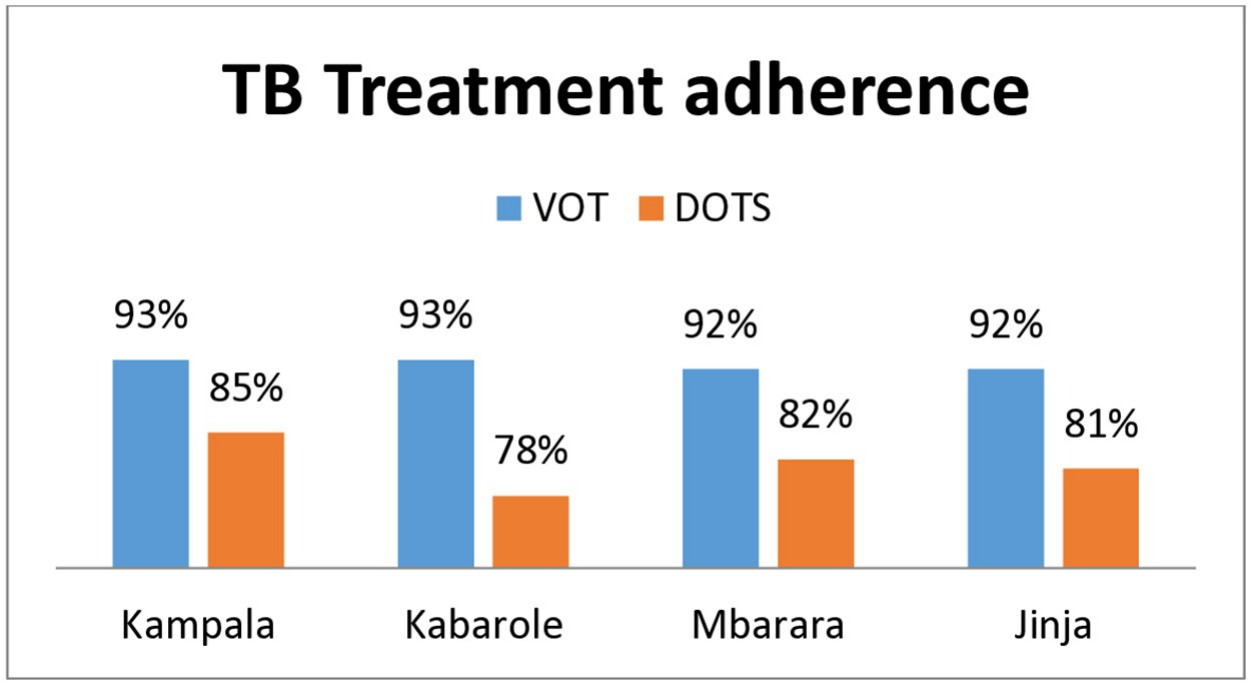
VOT vs DOTS TB treatment adherence rates.

**Table 4:** DOTS vs VOT based data comparison in the 4 districts of Uganda.

Region	DS-TB Patients		Number of Treatment completion		Number of Cured cases		Number of Died patients		Number of Lost to follow-up cases	
	DOTS	VOT	DOTS	VOT	DOTS	VOT	DOTS	VOT	DOTS	VOT
Kampala	1046 (45%)	250 (25%)	858 (45%)	201 (24%)	684 (43%)	185 (24%)	80 (34%)	7 (30%)	76 (53%)	9 (22%)
Kabarole	335 (14%)	250 (25%)	267 (14%)	205 (24%)	230 (15%)	190 (24%)	32 (14%)	3 (13%)	10 (07%)	12 (29%)
Mbarara	453 (19%)	250 (25%)	365 (19%)	215 (25%)	314 (20%)	194 (25%)	80 (34%)	10 (43%)	36 (25%)	11 (27%)
Jinja	495 (21%)	250 (25%)	409 (22%)	225 (27%)	346 (22%)	213 (27%)	43 (18%)	3 (13%)	21 (15%)	9 (22%)
**Total**	**2329**(100%)	**1000**(100%)	**1899**(100%)	**846**(100%)	**1574**(100%)	**782**(100%)	**235**(100%)	**23**(100%)	**143**(100%)	**41**(100%)

**Table 5:** Treatment outcomes of DOTS & VOT adherence modalities compared.

Outcome	Adherence Modality		Chi-Square Value	*P* value
	DOTS	VOT		
**Treatment Completed**	1899	846	4.5371	0.033
**Treatment Not Completed**	430	154		
**Cured**	1574	782	43.87	0.001
**Not Cured**	325	64		
**Died**	235	23	59.38	0.001
**Survived**	2094	977		

### B. From the qualitative data analysis

Based on the findings, there is a lack of knowledge and awareness about tuberculosis in the community, especially among women. Participants revealed only basic information about the disease, and many relied on partial and ill-conceived knowledge. Limited communication tools, such as posters or information materials were found, contributing to the potential spread of misinformation. Regarding symptoms, common symptoms such as fever, fatigue, loss of appetite & weight loss were known by many, but not all; knowledge gaps exist for other serious symptoms such as blood in sputum or enlarged lymph nodes. Also, there were several myths and misconceptions and knowledge gaps on the facts like, the modes of spread of TB, whether TB is curable or not, duration of treatment of TB, infectivity status of a TB patient on treatment, do’s and don’ts for care givers of patients etc.

Public services were preferred because of their affordability and perceived social support, although some faced challenges in accessing public facilities. Community members lacked comprehensive knowledge about what to do and what not to do for TB patients, and common reasons for delaying or discontinuing treatment included stigma, delayed test results, travel issues, lack of social support and living conditions. Stigma and discrimination associated with TB, especially for women, was prevalent, resulting in social discrimination and mental health complications. Participants emphasized the importance of a balanced diet with specific foods during TB treatment.

Participants opined that using VOT is a better adherence mode compared to DOTS. Participants cited resource-saving benefits, including time and money, and appreciated the interactive learning embedded in VOT that enhanced their knowledge. They opined that VOT was best suited for all times, more so, during the Covid-19 pandemic wherein there were restrictions for travel and movement and also lockdowns and quarantine. During the Covid-19 pandemic, the participants mentioned that, the VOT mode of adherence was a boon to them, as they were able to get in touch with the Health Care Workers (Village Health Teams) online, get their doubts clarified, request for medications, were getting alerts and reminders, and all this at no cost. The participants of the FGD were very much in favour of the VOT mode of adherence, as it improved confidentiality, helped them save resources in terms of time and money needed for visiting a Health Centre or a Health Care Worker, had in-built features like alerts and reminders if they were about to miss a dose and provided them with health awareness messages in relation to nutrition during treatment, duration, dosage etc.

## Discussion

The ACTS model is integrated with Behaviour Change Communication (BCC) tools, encompassing digital stories on TB, educational modules about TB, interactive games, interactive posters, and self-learning tools. These BCC tools play a pivotal role in enhancing patient knowledge about TB, promoting timely medication adherence, and encouraging the adoption of correct health-seeking behaviour. These BCC tools are made in local language and locally relevant graphics. This personalized approach not only connects more deeply with the community but also makes the stories and content more relatable to the individuals receiving the information.

The Pre-& Post-test conducted on awareness and knowledge about Tuberculosis, its causes, treatment, highlights that creating awareness significantly increases knowledge and improves health seeking behaviour. It also promotes better adherence to treatment and in turn better treatment success rates and cure rates for TB. This finding is similar to other previous studies done elsewhere in Uganda and in other countries as well [24–28].

The comparison of VOT vs DOTS based adherence in our study, very clearly shows that VOT based adherence has better outcomes in terms of increased cure rates and decreased death rates of patients. Similar results have been reported in a multicentre, analyst-blinded randomised trial conducted by Story A., Aldridge R. W., Smith C. M. et al. [29] and a Mixed Methods Study assessing digital monitoring technologies in Uganda [30]. The VOT provides evidence-based reporting and a patient-centric approach, enabling patients to conveniently submit their adherence information to their treatment provider and reduce the burden of daily travel expenses. It also empowers TB patients to take full control of their treatment and self-manage it. To ensure privacy, patients’ adherence videos are encrypted, with only their assigned treatment supporter having access for review. The feasibility and acceptability of VOT for supporting and monitoring adherence to TB treatment has already been tested in pilot settings in Uganda [30]. Our current study results have only created a more robust evidence base in relation to use of VOT for large scale project monitoring. However large scale country wide implementation might require cost-effectiveness and cost-efficiency studies of the overall project implementation costs of using VOT.

The first four stages of the QAFF ACTS model form the core of this research study, as they emphasize the significance of patient empowerment and community involvement highlighting the bottom-up approach in managing TB effectively. By effectively using technology (digital story-telling, mobile apps, VOT based adherence), providing user-friendly tools and interventions, this model aims to enhance patient autonomy and engagement in their own TB treatment, ultimately leading to improved treatment adherence and outcomes.

During the Active Ground Building exercise, the Field Team faced several challenges including existence of multiple local languages and dialect, addressing myths and misconceptions in the Community, travelling long distances etc. Added to this was the Covid-19 pandemic due to which there were several travel restrictions and as a result of lockdowns, contact between the Health Care Worker and the patient decreased. The VOT also acted as an opportunity during the Covid-19 pandemic as the Health Worker was able to contact their designated patients and vice versa, so that there was a continuity of care in the TB treatment and also health awareness, inspite of the stringent lock down situations prevalent across the country during that time. To address the issue of multiple local languages & dialect, QAFF has developed the learning material including the digital stories in several leading local languages of Uganda.

The VOT was an opportunity to tackle the issue of meeting the households and patients for TB treatment adherence. Using VOT, the patient’s adherence was monitored from a distance. As per the WHO Tuberculosis Report 2022, during the Covid-19 pandemic there was little difference in the WHO African Region, where disruptions related to COVID-19 have had little impact on the number of people diagnosed and officially notified with TB, whereas in other WHO Regions, the number of people being diagnosed and officially notified has had a significant fall [5]. In contrast to this, insights from our Focus Group Discussions highlighted the plight that some of the TB patients had, in collecting their regular anti-TB medications, visiting the Health Centre for a follow up visit etc. During the peak Covid-19 pandemic period, transportation was also an issue and maintaining social distance etc. all lead to a dip in TB case identification and treatment. At this juncture, the participants of our Focus Group Discussion, appreciated the usefulness of VOT based adherence, wherein, they used to get regular reminders and also follow up from a Health Care Worker and last but not the least, they were able to get access to a Health Care Worker through a phone call from being at the comfort of their homes and were able to get guidance on other basic health matters as well (as in Covid-19 vaccination, relevant health messages etc.). Getting valuable information on Covid-19 vaccination, other preventive measures of Covid-19 disease, treatment modalities for Covid-19 were all collateral benefits on the ground for the patients that were in constant touch with the Health Care Workers through the VOT mode. Hence although the VOT was a very crucial mode of treatment adherence mechanism during the Covid-19 pandemic, it emerged as a sustainable bottom-up approach for TB treatment adherence monitoring for the time to come, irrespective of the Covid-19 pandemic.

The ACTS approach was cost effective to the final beneficiaries (as in TB patients and communities) in many ways. It helped patients to do daily adherence reporting, follow-ups for refill of medication, test remotely without the need to travel to Health facilities and bear loss in their daily wages. For the Health System, the almost live data availability empowers them to focus on low-performing areas and not waste resources on those that are performing well. The data also provides insights into communities’ behaviors. So it was a win-win for both the TB patients, Communities and the Health System. Similar perspectives were obtained in a recently published study done on Digital Adherence Technologies (DAT) by Leung CL et. Al. in Philippines [8].

A Report of the Uganda Digital Adherence Technology Assessment, an independent evaluation of the DATs in Uganda towards TB treatment, highlights some of the benefits and challenges of DATs [31]. The Report delves on the benefits in terms of access to HCPs by the patients, better access to medications, alerts for taking medications etc. In terms of the challenges, the report talks of the limitation in terms of availability of smart phones, access to internet, digital literacy etc. But it is obvious that, most of these limitations and challenges are being addressed and are getting better by the day.

In fact, as part of our pilot study and implementation research, to ensure access of smart phones to TB patients, two provisions were made:

a. Introduction of Device Loaning (without any charges to the patients) for those who wish to be on VoT, but do not have a device to support it. These devices were collected back after completion of the treatment.
b. Introduction of Group VOT, which enabled a family member or a community member do daily adherence reporting of 5–10 patients in a group in the vicinity. S/he also provides digital communication to strengthen and boost patient’s morale.

However, the above options need a more in-depth study and analysis in terms of issues related to scale up and sustainability.

The acceptance of ACTS approach in the semi-literate and low literate communities and also among communities of the lower socio-economic status, that we surveyed, is very encouraging. Active Ground Building tools and approaches lead to more case notification. The patient’s digital connect for VOT adherence reporting and advisories, substantially improved patient treatment outcomes and reduced fall outs. These results of ours are similar to some of the other recently reported studies [8,31].

The QAFF ACTS Model will have huge societal impact as it gives control to patients to manage their treatment using mobile phone tools with compliance reporting, reminders and other information channels. It helps in creating new networks of treated patients as new knowledge providers, who will serve as peer-educators in communities to provide assistive support to patients thus making it a Bottom-up, Community-led model.

## Recommendations and way forward

The QAFF ACTS Model which includes Active Ground Building, Active Case Finding, Active Patient Compliance, Active Community Led Supervision, Active Treatment Management and Active Ground Assessment seems to be a sustainable approach for creating health awareness related to TB amongst the population, identifying more and more TB cases from the community, treatment adherence monitoring, improved TB treatment success rates and thereby better cure rates, all being done as a Bottom-up sustainable approach, with keeping the TB patient and the community as paramount and central to the approach.

There has been a keen interest amongst the Leadership of NTLP, Uganda, to pilot the ACTS strategy at a much larger scale within other districts of Uganda and also to integrate the same into the NTLP programme. There have been requests from some of the neighbouring countries to adopt the ACTS Model and integrate the same in their National TB Control Programmes as well.

A cost effectiveness and cost efficiency analysis of the ACTS Model is also on the cards, as it would provide an evidence base for scaling up of this bottom-up, community centric approach to larger populations and finally integrate the same with the mainstream health programme of the country. The initial tentative cost analysis done by us indicates that, although there will be a slight increase in the operational costs of the current programme that is using DOTS to upgrade to the ACTS Model and the VOT based adherence mechanism, once the software platforms are designed and put in place, the benefits of the Model in terms of the increased awareness created and increased no. of lives saved as a result of better treatment adherence rates and cure rates, far outweigh the minor additional cost factor involved. This should rather be looked at as an initial investment that the country is making in strengthening their Health Systems, in order to reap rich dividends in the future years to come. Also, it is obvious that, some of these costs are on a higher side, when done on a pilot basis, whereas, once they are scaled up for mass deployment, the costs can actually be lowered, as per the Economies of Scale. Anyways, a detailed Costing Analysis is mandated for better clarity and decision making.

## Recommendation

- Large scale validation of the pilot study results,
- Implementing with the Government National Programme & phase wise scale up in all districts of Uganda,
- Scale up to other East African countries / African Union countries.
- Costing studies of the Model to be conducted–to prove cost efficiency and cost effectiveness

## Conclusion

Active Ground Building plays a significant role in creating more awareness amongst the community members, thereby helping in better treatment seeking behaviour, improved treatment rates, treatment adherence rates and in turn better cure rates. Video Observed Therapy (VOT) is far more superior than the DOTS strategy as a strategy for TB treatment adherence and VOT decreases the resources required in terms of human resource, time and money and is also a more sustainable mode of treatment adherence.

## Limitation

The Study was not able to compare the VOT adherence data to overall baseline DOT data of the districts as the data was not available in the same format. All the other parameters as in education status, socio-economic status of the community members using DOTS was not available for comparison.

As a way forward, one of the future recommendations would be to plan the study in such a way that the DOT data in the districts is considered as the baseline data and a comparison of the same would be done with the VOT related adherence data.

## Supporting information

S1 File Annexure 1. (DOCX)

S2 File Annexure 2. (DOCX)
