# Supplementary material for: A community based, bottom-up, multi-pronged, technology integrated approach to enhance tuberculosis related awareness and treatment adherence in Uganda: The ACTS model
Source: PLoS One. 2025 Feb 18;20(2):e0318174. doi: 10.1371/journal.pone.0318174 (PMC11835331; doi:10.1371/journal.pone.0318174)
Supplement: S1 File — (DOCX) [file pone.0318174.s001.docx]

**Annexure 1:**

Structured Questionnaire used for the Pre and Post Active Ground Building Exercise

**Structured Questionnaire**

| **Pre / Post assessment questions** |
| --- |
| 1. What causes TB?  - Don't know - Bacterial infection - Smoking or drinking too much - Working in unhealthy conditions  1. What part of the body does TB affect?  - It can affect any body part - Lungs only - Not sure  1. Most common form of TB?  - PTB - EPTB - Don't know  1. What are the three common symptoms of PTB?  - Fever - Loss of hearing - Night sweats - Persistent cough more than 15 days - Lymph node swelling - Weight loss - Other please clarify - Not Sure  1. Do you remain infectious until you complete your medication?  - Yes - No - Mostly but not always - Don't know  1. Do you know how PTB is diagnosed?  - A doctor examines you - You have to get an x-ray - Sputum testing - Other please clarify - Don't know  1. Is PTB curable?  - Yes - No - Mostly but not always - Don't know  1. Do you know how long PTB treatment lasts?  - 12-24 months - 6-8 months - 8-12 months - Not sure  1. Will PTB affect your biological ability to have children?  - No - Yes - Don’t know |
