# Supplementary material for: A community based, bottom-up, multi-pronged, technology integrated approach to enhance tuberculosis related awareness and treatment adherence in Uganda: The ACTS model
Source: PLoS One. 2025 Feb 18;20(2):e0318174. doi: 10.1371/journal.pone.0318174 (PMC11835331; doi:10.1371/journal.pone.0318174)
Supplement: S2 File — (DOCX) [file pone.0318174.s002.docx]

**Annexure 2:**

**Topic Guide for the Focus Group Discussion (FGD)**

**Focus Group Discussion Guide: Exploring Community Perspectives on Tuberculosis**

| 1. **Group No.: ____________________________** | |
| --- | --- |
| 1. **Date: _________________________________** | |
| 1. **Village, District: ________________________** | |
| 1. **No. of Participants** | **Male: _____________** |
|  | **Female: ___________** |

**Please record responses in text and audio forma**t.

Introduction: Welcome and introduction to the purpose of the discussion.

**Rapport building Questions.**

**Themes for Discussion:**

1. About Tuberculosis
2. Symptoms
3. Transmission
4. Method of Prevention & Control Spread of TB
5. Public Treatment v/s Private Treatment
6. Do and Don’ts for TB Patients
7. Common Reasons for Delaying TB Treatment or Leaving Treatment in Between
8. Importance of TB Treatment Completion
9. Stigma & Discrimination Related to TB
10. Diet/Food Suggestions
